# Notes on Medical Matters and Medical Men in Paris

**Published:** 1847-02

**Authors:** David W. Yandell

**Affiliations:** Louisville, Ky.; Paris


### WESTERN JOURNAL

OF

### MEDICINE AND SURGERY.

FEBRUARY, 1847.

---

**Art. I.**—*Notes on Medical Matters and Medical Men in Paris.* By David W. Yandell, M.D., of Louisville, Ky.

Of the living chemists of France the names which would occur first to an American, are those of M.M. Orfila and Dumas, the latter distinguished for his contributions to organic chemistry, and the former for the zeal and success with which he has studied the subject of poisons. I have had the pleasure of hearing both of them lecture. A fortnight ago I listened with great interest to a lecture of an hour and a quarter's length by Orfila, who, as you are aware, is professor of medical chemistry in the *Ecole de Médicine.* The room in which he lectured is an amphitheatre, capable of containing five hundred persons and upwards; the seats are without backs, and the whole appearance of the room is decidedly plain. It is said that more students attend Orfila's course than that of any other professor in the school. The morning on which I

attended him his room was crowded to excess. The rush of the students, who were not admitted until a few minutes before the hour of the lecture, was as great as that which has so often made the Institute building to shake again; but the shouts were not heard. The venerable, commanding, and intellectual looking professor made his appearance just as the clock struck half past ten, the hour for commencing his lecture, and was received by the class with considerable clapping of hands; I did not hear a foot stir. Orfila cannot be less than fifty-five years old, if he is not sixty; his head is bald over the whole of its upper half, while the back part and temples are covered with thick, nearly white hair. He is but a line under six feet in height, of fine form, and in every way a man of striking appearance. His head is ample and well formed, his eyes black, his nose Grecian, his mouth large and expressive. A better lecturer than Orfila one will not often hear on any subject. With his fine person, large, penetrating eye, clear, strong voice, and earnest manner, he makes an impression upon his hearers which does not speedily pass away. He is emphatically an earnest man; his countenance, voice, gesticulation all testify to that essential attribute of the orator. While lecturing he walks from end to end of his long table, his experiments being performed by assistants, of whom there were three the morning on which I attended. Three assistants were too many by two, at least for this lecture, and as a consequence some glass was broken, and one experiment was a failure. There are few things which too many hands do not spoil. The apparatus exhibited during this lecture was plain, and not as well kept as it might have been.

The lecture was professedly on oxygen, but half of it was devoted to electricity. Among the experiments pertaining to the latter subject, I may mention the dazzling one of igniting charcoal points by the electricity of a galvanic battery. This experiment succeeded well when performed in the atmosphere, but when attempted under water it could not be called any thing but a failure, which was owing to the awkwardness of one of the assistants, who did not bring the points properly in contact. The oxygen used in the lecture was obtained from the black oxide of manganese, and also from water by the action of the galvanic battery, as is now done in every course on galvanism. No experiment was performed which I had not witnessed before in Louisville, and all that Orfila did in this way was, simply to immerse a taper in the bottle containing the oxygen to demonstrate its presence, the rest being left to his assistants. At the close of every division of his subject the professor drew from his pocket a silver snuff-box, took a pinch of snuff, replaced the box, used his handkerchief, and then continued his discourse. I have repeatedly heard him since in his tri-weekly course, but, although always interesting, he has said nothing yet new to me, or which, I suppose, would be new to your readers. I promise myself great advantages from his lectures on poisons, a branch of chemical science in which he is unrivalled, and when he reaches that part of his course I may be able to report something of interest.

The students in attendance at the School of Medicine are a well dressed body of, generally, quite young men; among whom there are many who are well favored, but not a few who are the reverse; some are of good size, but many, the majority, are small. They sit with their hats on, are very attentive, and in Orfila's theatre, made no noise, except at the appearing of the professor, and at the close of his lectures, when there was a clapping of hands, but not a single stamp. I saw several students taking notes, and many more who would have done so if they could have commanded the elbow room. It would not be a bad plan to construct all lecture rooms after the fashion of those in London, with boards on the backs of the seats, which serve as desks for writing; for unless a student takes notes upon what he hears he is not likely to remember much, and if he postpones doing so until he reaches his room, besides the time which it would require to bring his notes up, he is not apt to feel much inclination towards the task. The hardest, and the best informed students of my acquaintance at home, in England, and in France, are those who are most industrious with their pens or their pencils.

Orfila paid a visit, not long since, to Spain, and while there wrote home some interesting letters concerning the state of medical science and medical education in that country. From one of these letters, which appeared a day or two ago in the *Gazette Médicale*, I learn that nine years are necessary to prepare a student for the title of Doctor of Medicine, and that he must study seven years before he is entitled to a diploma, which confers upon him the right to practice in Spain, but does not make him eligible to any professorship. To an American this appears a long time, and few among us, I presume, would willingly enter upon such a probation.

At the meeting of the *Academy of Sciences*, on Monday evening, the 25th of October, among the numerous *savans* present, I was particularly struck with the appearance of Dumas. In the long hall of the Institute of France, in which the Academy holds its meetings every Monday, is a table of an oval form, divided near the middle of one of its sides by the chair and desk of the President, which extends from one end of the room to the other; members sit in chairs on the inside and on the outside of this table, and in addition there are chairs and other tables in the space formed by the oval. The accommodations for visitors are not ample, consisting merely of two rows of benches arranged on one side next the wall, one row on the other side, a few chairs and a little space for standing at the ends of the room.

I took a seat near the entrance in order to learn the names of the members as they entered from the obliging man, whose business it was to stand at the door and prevent persons from coming in after the seats were filled. I was looking about at the different persons present when my cicerone touched me, saying, "there, sir, is M. Dumas." I turned, Dumas was bowing most gracefully to his acquaintances and confreres as he passed them on the way to his chair, at the left hand, and in front of the president. Seated, his appearance was striking, and his face and person agreeable. He is a young looking man, not seeming to be past thirty-five. In height he is about five feet ten inches, may be a fraction more or less; his hair is a glossy black, and towards its ends waved or inclined to curl; his complexion is dark; his head large; his forehead bold and high; his eyes, I should say, are dark, but whether grey or black, I was too far removed to tell; his nose is prominent and shows a tendency to the variety called 'hook;' his mouth, which is one of the most expressive features of his face, is well turned and emphatically that which belongs to a speaker.

His entire suit was black, save the white cravat which was barely seen above the collar of his velvet waistcoat, which buttoned straight up to his throat. The mark of the legion of honor, a red ribbon, was fastened to a button hole in the left lapel of his coat. He might have been mistaken for a young Episcopal divine. His voice is clear and resonant; his articulation distinct and sufficiently varied, and his manner while reading appropriate. I say while reading, because I only heard him read a paper. Of his style of lecturing, which I have heard so often and so highly praised, I shall acquaint you as soon as he commences his usual annual course early in the coming month. The subject of the paper, which he read before the Academy on that evening, was the conversion of sulphuretted hydrogen into sulphuric acid.

M.M. Humboldt and Boussingault have shown that the water of the Rio Vinagre which, near the volcano of Puraci, falls in a cascade of acidulous water, contains free sulphuric acid, as well as free chlorohydric acid. M. Dumas has made the production of sulphuric acid under these circumstances the subject of his investigation. He has established this general fact, that sulphuretted hydrogen mixed with air through the aid of porous substances, but especially of linen, and under the influence of a slightly elevated temperature, is slowly converted into sulphuric acid. This mode of combustion differs entirely from that which is observed when sulphuretted hydrogen is burnt in the atmosphere with the evolution of light and heat. In this latter case the products are water, sulphurous acid, and almost always a deposit of sulphur and traces of sulphuric acid. In the case of the slow combustion of the sulphuretted hydrogen, effected under the preceding conditions, M. Dumas has perceived that neither sulphurous acid nor sulphur have been produced, but that the only result has been the formation of sulphuric acid.

When, on the contrary, water containing sulphuretted hydrogen is exposed to the air, a slow combustion occurs here also, but water only is formed, and sulphur precipitated. The deposit which obtains in all sulphurous waters is a well known illustration of this. In many places he has had occasion to remark the sulphate of lime which had resulted from the action of sulphuric acid produced by the sulphuretted hydrogen. In all great cities, but more particularly in London, it has been observed that large masses of iron exposed to the air become eroded; and this change has been attributed to the presence of sulphurous gas in the atmosphere. This gas is supposed to result from the combustion of coal; though it is a question worthy of consideration whether the sulphuretted hydrogen exhaled from the numerous sewers does not contribute to produce the effect.

Sulphuretted hydrogen may slowly form sulphuric acid, and wherever there are substances upon which this may act sulphates result; thus according to M. Vogel and others, whenever alkaline sulphates are brought in contact with organic matter, sulphuretted hydrogen is produced; and whenever humid remains of plants are exposed to the action of sulphuretted hydrogen and the atmosphere, sulphuric acid and the sulphates will be re-formed. In the form of sulphates then, sulphur may travel through the air in its watery constituent to lands which need it either for the development of vegetables or animals. He thought the part played by yellow sulphur in the production of all the azoted portions of plants and animals was not unworthy of remark; on an average, the 100th part of their entire weight is sulphur. Thus ten kilogrammes of dry azoted matter, which is about the quantity in a man of ordinary size, contain one hundred grammes of sulphur. To extend the calculation, two millions of kilogrammes is the representative number of the amount of sulphur contained in the population of France, and twenty millions of kilogrammes is the equivalent of the sulphur contained in all the animal creation of France.

I wrote the foregoing some days ago, immediately after attending a meeting of the Academy. Monday, the 16th of November, at 1 o'clock, Dumas delivered the introductory lecture at l'Ecole de Médicine. The court in front of the doors leading to the lecture-room commenced filling at half past 9 o'clock in the morning. The day was cold, the thermometer standing at about 32°, and the students who had assembled so early were obliged to walk about briskly to keep themselves warm. The crowd gradually augmented, and at half past 11 large numbers of medical and other students took their places at the two doors. From this time until a few minutes before 1 o'clock the influx was rapid, and the multitude began to evince signs of impatience and ill humor. As the clock struck one the doors opened simultaneously and the rush was tremendous. I was in the front rank, and had I not been trained to such races before I might not have escaped as well as I did. As it was, I obtained a seat upon the second row of benches, in a position from which I could see nearly the whole audience, and very distinctly hear the professor.

As, I presume, is the case always in a Paris lecture-room where seats are so much in demand, there was jamming, sqeezing, bullying, cursing, and, what is so common among French students, *blackguarding* as we style it. Some tried to obtain places by sitting down in the laps of others, some by jumping in while others had risen up to see a contest going on in some other part of the room. The two doorways were full; the students who had been distanced in the race up stairs, now tried by pushing from without to precipitate those who were standing, upon the backs and heads of their fortunate companions seated below; nor were they entirely unsuccessful, for several men lost their balance and fell forward, creating such confusion that the lecturer was obliged to make more than one long pause in his discourse. Considering that from seven to eight hundred students were crowded into a room calculated to seat about five hundred, there was a most commendable degree of silence and order, which, in fact, were never disturbed, except when some corpulent man was forced to cry out for quarters, or some light youngster was pushed from his place, and tumbled headlong upon his neighbors below him. Amid all the scuffle not a blow was struck; one man was kicked at the close of the lecture, and the quarrels were numerous, but they ended, as all the quarrels that I have witnessed in Paris have, in the most disgusting scurrility.

Dumas and the other members of the Faculty wore the black and purple robes. The eloquent discourse was read, and consisted principally of a eulogy upon Augustus Bérard, who died not long since loved and lamented by all who knew him—a loss to science, to France, and the world. Dumas was at times touchingly eloquent, and more than once was obliged to pause from emotion, as he was repeatedly compelled to do by the plaudits of his audience. His lecture was more than an hour long; it has been published in the *Gazette des Hopitaux*, and after having read it carefully I am prepared to pronounce it a most beautiful discourse, abounding in noble thoughts, clothed in the richest drapery. The annual distribution of prizes was made at the close of Dumas' lecture. The gentlemen to whom they were awarded came forward and received them at the hands of Orfila, but the *sages femmes*, although their names were announced, were not seen.

*Clamart* is the name of an establishment for dissection, having large, well aired rooms for this purpose, besides a theatre for lectures, and a museum. The subjects are furnished by the hospitals. The museum contains comparatively but a small number of either anatomical or pathological preparations; but among these there is a considerable variety of curvatures of the spine, which are highly interesting and instructive. Among the preparations of the bloodvessels is one of irregular distribution of the arteries of the lower extremity, which may be regarded as not only unique, but important in its connection with surgical diseases and operations; unfortunately the specimen is not perfect, in as much as the aorta has not been preserved. One large arterial trunk follows the course of the posterior iliac, and furnishes to the pelvis the usual number of vessels supplied by that artery. The same great vessel then emerges from the sacro-sciatic notch a little to the outside of the deep or pelvian pudic artery; afterwards it pursues the course and accompanies the great sacro-sciatic nerve until they both enter the popliteal region, when its future connections and distributions are normal. You will observe that this great trunk represents, first the internal iliac, and afterwards the artery of the leg. It is natural to inquire in what manner the thigh was supplied with blood. On examining its anterior region, an artery of the thigh, the profunda, is seen, but its connection with the lower part of the aorta, from which evidently it had proceeded, is destroyed. This vessel, the profunda, appears to have followed the direction of the external iliac without sending off any vessels except the circumflex iliac and epigastric, until it had gained its usual situation in the thigh, when it divides in the customary manner. The internal circumflex is much smaller than common, and, as always occurs under such circumstances, the obturator is proportionally large.

Here is a case which I have watched at *La Charité*—it was gangrene of the left arm, and terminated fatally. The patient was a man 30 or 35 years old, a nailer by trade, had constantly enjoyed good health, and was never exposed to contact with animals or any portion of them, who had been affected with malignant pustule or *charbon*. The disease commenced with a small pustule over the deltoid muscle and was the seat of considerable anxiety, accompanied by severe itching which induced the patient to scratch until he broke it. He entered the hospital the fifteenth day of the disease; the pustule presented a black slough of one centimeter in diameter, and situated in the point first attacked. Immediately afterwards it increased much in volume, became indurated and surrounded by a phlegmonous circle. On the third day the whole limb was tumefied, red, hot, and painful. The swelling increased rapidly the two following days. Below the slough the limb was exceedingly tense and œdematous, especially in the region of the hand, and presented in different places livid spots.

The slough becoming gangrenous was cauterized with butter of antimony. The ensuing day the gangrene became more defined, and the wound was cauterized with the hot iron by M. Velpeau. This, however, did not arrest its progress; by this time it had extended almost as low as the elbow; the gravity of the symptoms continued unabated, shivering came on, followed by fever and other constitutional symptoms, with great anxiety in the precordial region, and diarrhœa. The limitation of the gangrenous eschar which now seemed to have occurred, induced M. Velpeau to amputate the limb at the shoulder joint, from which the patient appeared to suffer no pain. There was an apparent alleviation of the symptoms the first day; the pulse fell from 140 to 120 beats in a minute; but the day following difficulty of deglutition, a sense of oppression in the region of the heart, with cold, clammy perspiration, ensued, and soon the patient sunk, retaining his self-possession to the last.

The amputated limb when examined showed all the tissues involved by gangrene down to the bones. On inspection of the body nothing was observed except slight congestion of pleura costalis of the left side. A large fibrinous clot of unusual consistence occupied the right auricle and ventricle; the arteries and veins were unaffected. The existence of this coagulum is supposed to have been connected with the precordial symptoms which constituted one of the most prominent and unpleasant features of the case. This is a brief report, but contains all that it seems to me was presented by the unfortunate and interesting case. I may mention that M. Jobert of *l'Hôpital St. Louis*, has lately reported a case of malignant pustule which is intimately connected with the gangrenous affection just noticed, in which the use of the red-hot iron was followed by recovery.—Frictions of the tincture of iodine have been recommended in inflammations of the *os uteri*, by Dr. Licherer of Heilbronn.

*On the Division of the Tendon of Achilles.*—Prof. Stromyer has lately published the following propositions relative to the section of this tendon, which I find in the Gazette des Hôpitaux of the 12th of November:

1st. The separation should be performed with a small knife, the blade of which is slightly curved at the point and very sharp; the subcutaneous method should be employed, and but one opening made in the skin.

2d. The tendon must be completely divided or the operation will not be successful.

3d. When the other muscles, or the plantar aponeurosis are retracted simultaneously with the tendon, they must be divided before the section of the latter is made.

4th. After the operation, compressions are applied to the wound by means of the figure 8 bandage.

5th. Among adults on the fourth or fifth day of the operation, and among infants on the third or fourth day, the first dressing is removed, and if it is found, as is generally the case, that the little wound is healed, you proceed to make extension; this is never to be resorted to while the wound suppurates or any considerable degree of ecchymosis exists.

6th. The limb is bandaged and properly protected from compression, before the foot is placed in the machine for extension.

7th. Extension is performed gradually and gently, and diminished whenever the patient complains of pain.

8th. In order to avoid excoriations, erysipelatous inflammations, and mortification of the tissues, the dressings should be removed whenever the patient feels any severe or continuous pain in the points under pressure.

9th. When the dressing is removed the limb should be covered with flannel.

10th. It is known that individuals who have submitted to the operation in question experience a sensation of cold and numbness, which is sometimes limited to the heel, and in some instances extends throughout the whole of the foot; this sensation diminishes gradually, and disappears ordinarily on the sixth or eighth day.

11th. The first or second day of the operation, although the individual may not have been subject to sweating of the feet, the foot which has been operated upon gives off a viscous exudation of a disagreeable odor.

12th. In placing the foot in the machine for extension it must form a little more than a right angle with the leg, and be kept in this position for eight days. After this period the foot must be wrapped with a circular bandage, and the patient must be allowed to make no attempts to walk before the fourth week; without this precaution the member will become swollen, the cicatrix will be irritated, and perhaps even the newly formed tissues destroyed.

13th. It is impossible to define the exact duration of the cure; this will depend on the state of the patient, the degree of the deformity, and the extensibility of the articular ligaments.

Dr. Moij'Sisovics, first surgeon to the imperial hospital at Vienna, has addressed to the Academy of Medicine, at Paris, a work entitled "An account of a sure and speedy method of treating Syphilis, by the preparations of Iodine." The author wishes the Academy, through a committee, to investigate what he has announced, and make a report on his work.

M. Fantonetti, an Italian physician, in the treatment of *acute articular rheumatism*, administers, instead of the large doses of quinine used in some of the French hospitals, only two grains of the sulphate of quinine every two hours, associated with the same quantity of crystalized tartaric acid.

M. Gazzi, also an Italian physician, speaks in terms of praise of the efficacy of the black hellebore in melancholy and mania—the treatment of the ancients.

Valerianate of zinc has been highly recommended as an *anti-neuralgic*, by M. Narnias, who has witnessed its good effects in a number of cases.

M. Filacchione, in one of the late Italian journals, speaks of the nature and the treatment of the visceral alterations in autumnal fever. The visceral changes most commonly observed are in the liver, spleen, and pancreas, and are dependent, in the opinion of M. F., upon a lesion of the ganglionic plexus, which presides over the vitality of these organs. If there is merely a hyperemia of the liver, &c., he administers the sulphate of quinine associated with rhubarb, extract of gentian, and other bitters. If it should have gone on, however, to a hypertrophy, he has recourse to the muriate of lime, to calomel, with extract of aconite and taraxacum; covers the region over the affected organ with cataplasms of mallows flowers and hemlock, and gives as a drink decoction of the same flowers, with acetate of potash, and the syrup of chicoree and taraxacum. This treatment has always succeeded in preventing relapses. In cases where the consecutive alterations persist a long time, and are associated with serous collections in the abdomen, he uses mercurial frictions upon the abdomen; internally syrup of squills, united to colchicum and calomel, and enjoins the milk diet.

M. Cattell proposes to obviate the irritation of the digestive organs and the nephritic symptoms, which frequently follow the administration of copaiba and cubebs in the usual mode, by giving them in the shape of an injection.

M. Jobert, at *l'Hôpital St. Louis*, in one of his clinics, spoke of the causes and treatment of erysipelas. He believes that the cause of erysipelas exists in a peculiar constitution of the atmosphere, and that it is alike independent of the gastric conditions and traumatic lesions, though the former may render its progress more rapid, and is susceptible of facilitating its development, though incapable of producing the disease.

If it is asked why then does erysipelas show itself in patients who are suffering from wounds, first upon the injured parts? The question is easily answered. Why, in a number of persons exposed alike to the same vicissitudes of temperature, is one affected with pneumonitis, another with pleuritis, and the third with arthritis? We account for these differences by admitting a special disposition of the organs, a susceptibility more or less great, which renders them more or less liable to feel the effects of the same cause. This susceptibility may be either the result of a peculiar physiological disposition or tendency, or of a pre-existing pathological condition.

Every one knows that a person who has suffered often from bronchitis, acute articular rheumatism, &c., is constantly exposed to a repetition of the same diseases under the influence of the slightest causes. The same thing in reality occurs when erysipelas supervenes in a patient who, in some part of the body, presents a solution of continuity; if in such cases the eruption shows a preference for the borders of the wound, it is because at those points the skin has been the seat of an inflammation which, notwithstanding that it may have disappeared, has nevertheless augmented the susceptibility of the part, and by that means has rendered it more liable than any other portion of the body to take on erysipelas. There is scarcely any doubt that erysipelas often exists and produces certain constitutional modifications before it offers anything to the eye of the surgeon; and it is a mistaken idea, that wounds are the occasional cause of the affection.

Erysipelas has often prevailed as an epidemic in the wards of M. J., and supervened indiscriminately upon patients who presented wounds and those who presented none; so frequently, indeed, has such been the case, that he considers himself authorised to regard the atmospheric constitution as the principal cause of the disease. Among all the objections which may be urged against this theory, none will destroy the important and remarkable fact, that erysipelas and hospital gangrene are two complications more frequent in the wards of M. Jobert than in any other wards of the hospital, and that his ward, (that of the men), is more unwholesome, more poorly aired, in a word, that the patients in it are placed in the worst atmospherical conditions.

Epidemic erysipelas has prevailed in other epochs than our own. It was remarked by Tozzi to prevail in the autumn and winter of 1770, at Naples. In 1773 Wm. Bromfield made mention of an epidemic erysipelas, which continued two years. M. Velpeau published in the Gazette des Hôpitaux, in 1831, some observations on epidemic erysipelas.

Whatever may be the nature of the eruption, whatever may be its form, the treatment employed by M. Jobert is always the same, consisting of an ointment of nitrate of silver, which, though it may vary in strength, acts always in the same way; M. Jobert esteeming it a powerful antiphlogistic. He uses three species of ointment, differing in strength as one, two, three; the first contains thirty grammes of simple cerate, and four grammes of nitrate of silver; the second eight grammes; the third twelve grammes.

To the two forms of hospital gangrene, 1st, the ulcerous described by Delpech; 2d, the pulpy form, described by Dussaussay, for which, however, M. Jobert substitutes the albuminous form, since he considers that the term conveys a much more definite idea of the nature of the malady, this surgeon adds a third—the form *ramollissement.* This, which is not described in the books, is characterized by patches, varying in extent, gray, soft, sprinkled or studded with small black points, which appear to be formed by small ecchymoses. The granulations completely destroyed, they are themselves the seat of mortification, whilst, in the preceding form, you are able by a brush with the greatest facility to remove the fibrin which exposes to view the red granules more or less altered in organization, and that bleed when exposed.

The cause which seems to have most influence in developing hospital gangrene is the atmospheric constitution. Of the three varieties the second, or albuminous, is incontestably the most frequent.

The treatment used by M. Jobert is to dress the wound with camphorated spirits or lemon juice; if the disease continues, the parts covered with the albuminous matter are cauterized with the nitrate of mercury. Should the malady pass its first stages, M. Jobert resorts to cauterization with the red-hot iron.

M. Labus, an Italian, having established the diagnosis of Bright's disease, in a woman aged 32 years, gave as a drink a mixture of four grammes of medicinal nitric acid, in seven hundred and fifty grammes of water, with simple syrup and mucilage of gum arabic, each fifteen grammes. This drink was first administered on the 16th October, it was discontinued on the 26th January, 1846, when the urine was normal, her nutrition excellent, and the embonpoint restored.

M. Scotti, another Italian, having remarked that in some cases of scrofula which he had treated with the preparations of the juglans regia a habitual constipation resulted, conceived the hope of finding this agent a remedy against diarrhœa. It is applicable to all cases of diarrhœa except when the disease is accompanied by inflammatory symptoms. M. Scotti prefers the extract obtained from the hull of the walnut, and the green leaves of the tree. He dissolves eight or twelve grammes of this, which he has obtained by decoction and evaporation, in a kilogramme of mineral lemonade, and administers a third or a half glass full of this drink four times a day.

One of the medical societies of Paris excludes from becoming its members all those who, in any way, countenance charlatans.

I do not attend with any regularity the meetings held on every Monday, of the Academy of Sciences, because if I would procure a seat I must stand by the door for an hour and a half before it is open, and that is more time than I can spare, and requires more patience than I can muster. The meetings are not generally of especial interest, more particularly after one has attended one or two and seen the principal men, as I have done.

M. Roux, at *Hotel Dieu*, is an old, short, thick, and not intelligent looking man, who speaks with great rapidity and little distinctness, though his clinical lectures are replete with good sound principles, so far as I, who understand the French so poorly unless when it is spoken slowly, can judge.

A week ago he ligatured the femoral artery of the right side for an aneurismal tumor situated at the superior extremity of the tibia, that is to say, in the spongy tissue which constitutes the tuberosities of this bone. Before performing the operation he spoke at length of aneurisms occurring in both the soft parts and in the bones, but more particularly of the disease in question. But to give you a regular outline of his valuable lecture.

Diseases of this kind belong to the great class of *Angiectasis*, tumors formed by the blood, and resulting from a dilatation of the vessels. The angiectases form two distinct categories; those which appertain to the large vessels, and affect most frequently one vessel alone; and second, those which have their seat in the capillaries, and result from an enlargement of these vessels, and a change of their nature and consistence of the parts which surround them. The name of *tumeurs fongueuse sanguines*, which the French render *fungus hematodes*, may be translated erectile tumors, aneurism by anastomosis, certain tumors formed by the accidental development of a spongy or alveolar tissue containing a large quantity of blood, and appearing to result from an inextricable interlacing of dilated and altered capillary vessels, &c., &c. Along through the discourse M. Roux called this a *tumeur sanguin*, or bloody tumor. He made another division by which those tumors (*tumeurs sanguines*) were classed —those which occur in the soft parts, and those which are seen in the skeleton. The difference between them is very great, both in the causes of their production, their seat, &c., &c.

For the fungous tumors of the bones there are three methods of treatment; 1st, the ligature of the principal artery of the member; 2d, amputation of the member itself; 3d, in rare cases, the removal of the portion of the bone which is the seat of the disease. In Roux's long practice, with an experience of thirty years in hospitals, he has only observed this form of the affection (fungous tumors of the bones) three or four times. It is a disease which may be developed in any bone of the skeleton, provided it possesses a spongy tissue, but it is much more common in the bones of the extremities, and in those of the inferior rather than of the superior. It is encountered almost entirely at the articular extremities of the bones, which it is well known are the portions most abundantly supplied with spongy tissue, and the tibia is both by its structure, organization, nature, and disposition of the tissue, &c., especially exposed. It is produced by accidental causes, as external violence, contusions, violent distension, or in some cases by a mere strain of the articulation. In the present case it supervened upon an attempt to relieve a carriage, in which the man brought his right knee into violent and prolonged action, after which he felt in the member a sense of distension as from a strain. When a blow or a fall has provoked its development, a longer or shorter period may intervene between the accident and the appearance of the disease. In cases where it follows another series of causes, as, in lifting weights, or straining the knee in any other way, the patient feels generally a cracking in the articulation, a distension, which as you have seen in this case, may occur suddenly.

The disease is noticed more frequently among young subjects than among adults. The appearances presented by the affected limb now before us, are, the superior extremity of the tibia is larger than that of the opposite limb; the leg has lost somewhat its natural form. The external lamellæ of the bone have been entirely destroyed, the destruction having commenced from within and come outwards, and through this pathological opening a portion of the alveolar, spongy, fungous tissue, which results from the fungous transformation of the bone, protrudes, forming a hernia—elevates the skin, and forms a more defined tumor. Placing your hand upon the surface of the tumor, you are able distinctly to feel pulsations synchronous with those of the heart, and which may be compared to those that you distinguish when you have an aneurismal tumor, except that you feel neither the force nor the extent of the pulsations which you do in pure aneurismal tumors. You feel the beats in only a small portion of the fungous tissue, and these are not very strong. The cause of these pulsations is well known.

At *Hotel Dieu*, in the wards of M. Louis, a woman presented herself laboring under erysipelas of the face. She was treated for this affection until all external evidences of the disease had entirely disappeared. She however continued extremely feeble, soon began to grow worse, and laboring under the symptoms of gastro-intestinal inflammation, she died. The autopsy revealed a lively red appearance of the mucous membrane of the stomach, and a part of the intestinal canal, and partial softening of this membrane in some portions of the stomach. The heart, lungs, etc., were healthy.

Dr. Boudet speaks in high terms of the efficacy of iodide of potassium, in the form of ointment, in articular rheumatism. He has seen a violent case of lumbago relieved in one hour under the influence of frictions with iodide of potassium, and disappear completely the third day. Rheumatismal inflammation of the thigh has been not less successfully treated in the same way; and M. Boudet proposes now to try it in inflammatory rheumatism of the articulations.

The administration of arsenic in the treatment of furuncles has been suggested and praised by Dr. Schweich, of Neurried. This physician prescribes four drops of Fowler's solution, morning and evening, until the patient has taken three grammes; five drops are now administered until other three grammes are consumed, when the dose is augmented to six drops, and persevered in until three more grammes are taken, which always succeeds in curing the disease. During this medication it is not necessary to change the ordinary diet of the patient. In the first week of the treatment it is not rare to see new furuncles appear, which however remain in their rudimentary state, and finally disappear. Relapses, after the use of this medicine, are extremely rare.

Among the thirty or forty cases of typhoid fever which have been treated during the last four months at *La Charité* by M. Briquet, only three have died. The treatment which he pursues is briefly this—an emetic of one or two grains of tartarized antimony; in the evening of the same day, a bottle of Seidlitz water, or a purgative injection. The second day a bottle of Seidlitz water, taken by half glasses during the day, in the guise of the ptisan. If this does not act sufficiently upon the bowels, you assist the laxative taken by the mouth, by a purgative injection. It is rare that purgatives have to be persisted in for any length of time; more generally in a very few days the febrile phenomena disappear, the pulse falls, &c., and in a very short time the patient is able to take a small quantity of broth. In typhoid fever thus treated you will remark in particular, that the convalescence is very rapid, and that it is rarely retarded by relapses; if, however, any should occur they easily yield to a bottle of Seidlitz water; finally, a very few days after the amelioration has begun, the alimentation may be composed of meat soup, or of meat itself.

M. Guirard, at *Hotel Dieu*, has prescribed sulphate of quinine in rheumatism, with very great success.

Here, perhaps, I may as well close this rambling letter. It will be remarked, I trust, that my endeavor has been to communicate as much matter in as little space as possible, without much respect to style. If the readers of the Journal have the patience to follow me through these desultory communications, I think I am safe in promising that my stay in Paris will enable me to add, each month, to their interest.

Paris, November 30th, 1846.

